# Stillbirth and newborn data quality and use and related input and process factors: findings of the IMPULSE study in Uganda

**DOI:** 10.7189/jogh.16.04153

**Published:** 2026-05-29

**Authors:** Ronald Wasswa, Lorenzo Giovanni Cora, Rornald Muhumuza Kananura, Peter Lochoro, Richard Mugahi, Jimmy Ogwal, Chris Ebong, Firehiwot Abathun, Dawit Fisshea, Jacqueline Minja, Mary Ayele, Ousman Mouhamadou, Ilaria Mariani, Francesca Tognon, Joy E Lawn, Giovanni Putoto, Donat Shamba, Peter Waiswa, Marzia Lazzerini

**Affiliations:** 1 Makerere University School of Public Health, Kampala, Uganda; 2 Institute for Maternal and Child Health IRCCS Burlo Garofolo, WHO Collaborating Centre for Maternal and Child Health, Trieste, Italy; 3 Demographic Dynamic and Population Health Unit, African Population and Health Research Center, Dakar, Senegal; 4 Doctors with Africa CUAMM, Kampala, Uganda; 5 Ministry of Health, Kampala, Uganda; 6 Doctors with Africa CUAMM, Addis Ababa, Ethiopia; 7 Ifakara Health Institute, Ifakara, Tanzania; 8 Doctors with Africa CUAMM, Bangui, Central African Republic; 9 Doctors with Africa CUAMM, Padua, Italy; 10 London School of Hygiene & Tropical Medicine, London, UK

## Abstract

**Background:**

The reduction of preventable newborn deaths in low- and middle-income countries is a global priority. The availability of high-quality newborn and stillbirth data is essential for shaping action-oriented policies and interventions towards resolving these challenges. Using a mixed-methods approach, we evaluated the input and process factors hindering data quality and use in Uganda.

**Methods:**

We conducted a cross-sectional study from November 2022 to September 2023 across three regions and one city administration in Uganda (51 sites, 30 facilities, 20 district health offices (DHOs), and a Ministry of Health). We collected data primarily through direct observation based on standardised Every Newborn – Measurement Improvement for Newborn & Stillbirth Indicators tools and analysed them using the Performance of Routine Information System Management framework. We synthesised data quality, data use, and their technical, organisational, and behavioural determinants using sub-domain level indicators designed to provide a novel approach for policymakers.

**Results:**

Newborn data availability and completeness were high, with denominator elements exceeding 90% at all levels and numerator elements at facility level ranging from 86% to 100%. In contrast, data accuracy was consistently low (range = 26–61%), and data use for performance review remained limited, particularly at facility level (21–69%) compared to DHOs (33–81%). Among underlying factors, key strengths included that most sites had staff to compile and analyse data (93–100%), used data visualisations (93–95%), and showed strong technical and behavioural performance at the DHO level. Conversely, promotion of evidence-based decision-making was low (58–64%), and critical resource and capacity gaps persisted, including limited availability of minimum item bundles (22–24%), functional internet (60–83%), staff development plans (55%), user skills at facility level (20–61%), and data analysis and feedback mechanisms (50–74%). Overall, 350 (74%) end users reported a need for improvement, with no significant differences across site levels.

**Conclusions:**

These findings provide actionable guidance for policymakers to improve the quality and use of newborn and stillbirth data. Emerging priority actions include strengthening the data verification processes, building analytical capacity at facility level, and institutionalising regular data review with focus on decision-making.

The reduction of preventable newborn deaths remains a priority globally, but particularly in low- and middle-income countries (LMICs), where neonatal mortality and morbidity rates remain unacceptably high [1]. An estimated 2.3 million newborns die each year worldwide, with sub-Saharan Africa accounting for half of these deaths [1]. Achieving further reductions in neonatal mortality requires not only effective interventions, but also accurate, timely, and actionable data to guide implementation and ensure accountability towards the Sustainable Development Goals, particularly Goal 3.2 [2–5]. Global initiatives such as Every Newborn Action Plan (ENAP) and Ending Preventable Maternal Mortality, now aligned under the Every Woman, Every Newborn, Everywhere Plan [6], have set ambitious targets to reduce neonatal mortality and stillbirths [7–9]. These efforts highlight the need for robust data systems to track progress and inform policy decisions [10–12].

Routine health information systems (RHISs) play a key role in tracking the coverage and quality of maternal and newborn health services delivered in health facilities [13–15]. This role has become increasingly important as facility-based deliveries have expanded globally, with over 80% of births in sub-Saharan Africa now occurring in health facilities [7,16]. However, despite the increasing reliance on RHIS data, substantial challenges persist in terms of data quality, availability, accuracy, and use, particularly in resource-constrained health systems, where competing priorities and limited capacity affect routine data processes [17,18].

The Performance of Routine Information System Management (PRISM) framework conceptualises RHIS performance as the result of interactions between technical, organisational, and behavioural determinants, alongside data management processes [19] (Appendix S1 in the **Online Supplementary Document**). Uganda adopted the District Health Information Software 2 (DHIS2) as its national RHIS platform in 2011 [20]; since then, several assessments have examined aspects of RHIS performance [21,22]. However, these studies have mainly focused on selected reporting dimensions such as completeness or timeliness, did not include underlying determinants, and did not focus on newborn specifically. As a result, data accuracy, system-level determinants, and end-user interaction with newborn and stillbirth data for decision-making across health system levels remain insufficiently examined.

The IMProving qUaLity and uSE of newborn indicators (IMPULSE) phase 1 study was designed to address these gaps across four African countries (Uganda, Tanzania, Ethiopia, and the Central African Republic). It assessed stillbirth and newborn data quality, data use, and a broad set of underlying technical, organisational, behavioural, and data management factors using direct observation, while also collecting end-user perspectives (**Box 1**). Here, we present the findings of the IMPULSE phase 1 study in Uganda with a novel reporting format based on item-level indicators aggregated into domain-level measures aligned with the PRISM framework, and end-user perspectives integrated into the analysis [19]. Our objectives were to:

Box 1 **Key findings**

What was known before this study?

- High quality data and high data use are needed to prioritise and monitor interventions aimed at reducing preventable newborn mortality and morbidity.
- Previous assessments highlighted major gaps in the completeness, timeliness, and accuracy of newborn and stillbirth data reported through DHIS2 in Uganda.

What did we find and what does it mean?

- Although several strengths were reported, substantial gaps in data quality and use were observed, specifically in data accuracy on all 10 newborn indicators assessed, and data use at facility level. Data completeness and availability at facility level were highly satisfactory, suggesting that good data quality is a reachable target for the current Uganda system.

What is new about this study?

- This is the first study documenting many determinants of newborn and stillbirth data quality and use in Uganda, and was carried out with a predefined, tested standardised methodology.
- Most data were collected through direct observation, and a series of quality assurance procedures were implemented.
- The synthetic reporting is a novelty aimed at offering a practical actionable high-level overview of findings, to favour interventions.

What is next for implementation?

- The results presented can be utilised for prioritising interventions tailored to the specific needs of each site.
- The applied methodology allows for replicability in other settings, or in the future in the same setting, to monitor results of any intervention implemented.

What research gaps remain?

- More implementation research is needed to further identify sustainable interventions to improve stillbirth and newborn data quality and use in Uganda.
- The PRISM methodology could be further optimised by identifying aggregate indicators that can be used in multivariate analyses.

− check the availability at national level of 16 ENAP-recommended indicators in DHIS2 in Uganda;
− assess the availability, completeness, and accuracy of 10 core and available routine newborn and stillbirth data elements across levels of the health system in Uganda;
− examine how these data are used for performance review and decision-making by end users;
− explore underlying the technical, organisational, and behavioural factors;
− report the staff perspective regarding the need for improvement in the electronic RHIS (eRHIS).

As part of a series of articles publish in this journal [23–27], our findings could inform prioritisation of actions to strengthen the whole system in the Central African Republic (CAR), Ethiopia, Tanzania, and Uganda, as well as similar countries.

## METHODS

As this study adopted a cross-sectional design, we report our findings per the STROBE guidelines for observational studies [28] (Appendix S2 in the **Online Supplementary Document**).

### Sampling and participants

**Figure 1:**
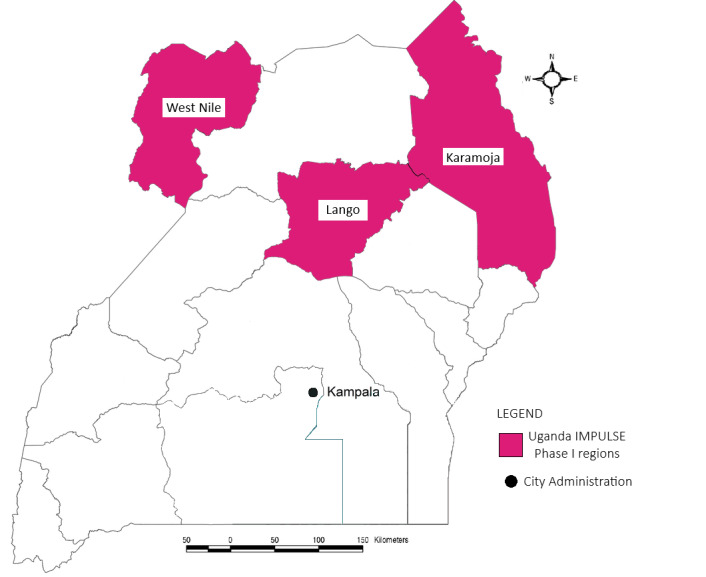
IMPULSE study map for Uganda’s regions and city administration assessed in this study. Regions included in the IMPULSE study are highlighted in pink. E – east, IMPULSE – IMProving qUaLity and uSE of newborn indicators, N – north, S – south, W – west.

The study was conducted in three regions and one city administration in Uganda (**Figure 1**), selected to ensure heterogeneity, feasibility of coordination through Doctors with Africa *Collegio Universitario Aspiranti Medici Missionari*), and alignment with Ministry of Health (MoH) priorities. Only comprehensive emergency obstetric and newborn care health facilities with or without neonatal inpatient care were eligible for inclusion. The sampling design (Appendices S3–6 in the **Online Supplementary Document**) was stratified to include facilities from different sites and levels. We selected higher-level facilities based on the number of deliveries, and health centres within the same districts for practicality. We also included the national hospital. Following the PRISM-adapted lot quality assurance sampling (LQAS) methodology [29], the minimum sample size was defined to be 19 health facilities.

### Data collection and quality assurance

We collected data from November 2022 to September 2023 using the Every Newborn – Measurement Improvement for Newborn & Stillbirth Indicators (EN-MINI), a tested newborn adaptation of PRISM tools, including ready-to-use digital data collection tools [29]. The EN-MINI-PRISM Tools were developed by the Every Newborn-Birth Indicators Research Tracking in Hospitals (EN-BIRTH) phase 2 research partners and further optimised and field tested in two countries (Uganda and Ethiopia) during the start-up phase of the IMPULSE study, in collaboration with the IMPULSE International Advisory Board, and the four IMPULSE national advisory boards (one for each country). Data collection and quality assurance practices are thoroughly discussed in other IMPULSE papers [23–27] (Appendix S7 in the **Online Supplementary Document**); all collection procedures followed the General Data Protection Regulation.

### Study variables, data stratification, and data analysis

Here, we analysed the availability at national level of key ENAP-recommended indicators in DHIS2 in Uganda); all indicators included in the PRISM User’s Kit except for indicators not directly defined as percentages [29] (387 variables, 187 for DHOs; 200 at facility level; Appendix S8 in the **Online Supplementary Document**); and six additional PRISM questions collecting the staff perspective regarding the need for improvements in the RHIS, in relation to newborn and stillbirth data.

**Figure 2:**
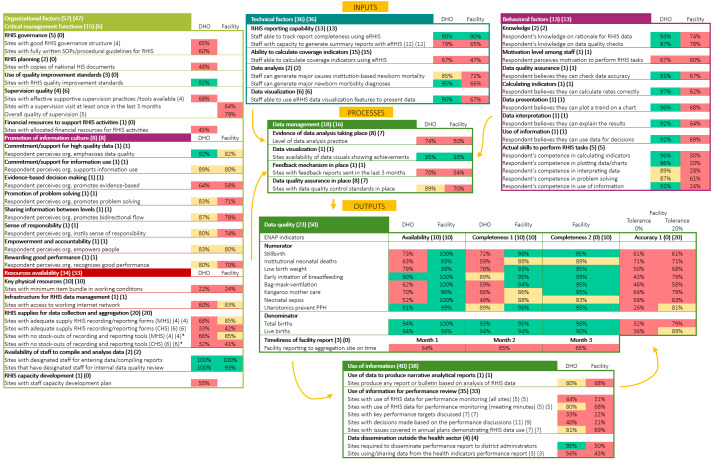
IMPULSE study: synthesis figure of the EN-MINI Tools Uganda assessment based of PRISM User’s kit [30] framework and conceptual model. Numbers in brackets next to each macro domain and sub-domain report the sample numerosity for DHOs and facilities, respectively. For completeness, ‘completeness 1’ is specific to the monthly reports and ‘completeness 2’ is specific to the completeness of source document. ‘Accuracy 1’ is specific to facility level. ‘Month 1’, ‘month 2’, and ‘month 3’ refer to the three months of data collection (April, May, and June 2022). ‘Facility’ includes all three level of health care facilities. Formulas are reported in Appendices S9–14 in the **Online Supplementary Document**. Colour coded as per the PRISM analysis tool: green marks the percentages between 90% and 100%, yellow indicates those between 80% and 89%, and red those below 80%. CHS – child health services, DHO – district health office, ENAP – Every Newborn Action Plan, EN-MINI – Every Woman Every Newborn-Measurement Improvement for Newborn and Stillbirth Indicators, eRHIS – electronic routine health information system,, HIS – health information system, IMPULSE – IMProving qUaLity and uSE of newborn indicators, MHS – mother health services, org – organisation, PPH – postpartum haemorrhage, PRISM – Performance of Routine Information System Management, RHIS – routine health information system, SOP – standard operating procedure. *In the last 6 months.

We analysed all data descriptively, summarising and reporting as per the PRISM User’s Kit [29] and further validating all analyses with the PRISM Automated Analysis tool [30]. We categorised data according to the PRISM framework [29] (Appendix S1 in the **Online Supplementary Document**) into macro domains (inputs (RHIS determinants), processes, and outputs) and corresponding sub-domains, while further stratifying them by site type (central MoH, district health offices (DOHs), and facilities). We presented data both in a synthetic actionable format of one page (**Figure 2**) and as detailed results (Appendices S9–14 in the **Online Supplementary Document**).

We calculated promotion of information culture and behavioural factors indicators as normalised PRISM scores, *i.e.* as the ratio between the scores obtained by the respondents and the maximum score obtainable multiplied by 100. We described central level (MoH) results as ‘presence/absence’ since they included only a single observation for most tools; for tool 6, two respondents were interviewed (Appendices S9–14 in the **Online Supplementary Document**).

Given the absence of a PRISM indicator for the physical resources [29], the IMPULSE team developed the indicator for ‘Key physical resources’, defining it as the percentage of sites with at least one of each of the minimum item bundle composed of calculators, computers, internet related items (*e.g.* modem), power related items (*e.g.* power supply, generators), and printers.

We calculated data accuracy among two key data sources (registers *vs*. DHIS2) via data reported over a three months period (April to June 2022), as per EN MINI Tools design, as the percentage of sites with perfect accuracy, defined as perfect data matching (*i.e.* ratio between the absolute records in registers vs those reported in DHIS2 equal to 1), and the number of sites with a data accuracy within ±10% (*e.g.* ratio between the absolute numbers reported in registers *vs*. those reported in DHIS2 from 90% to 110%). Only sites which delivered the health service were included in the denominator.

End users’ perspectives were first analysed for each tool individually and then summarised via weighted averages (weights based on the number of respondents of each tool). We compared statistical difference between proportions across site levels via z-test (Appendix S6 in the **Online Supplementary Document**).

An experienced statistician (LGC) performed all data analysis, with checks from a second statisticians (IM), in *R*, version 4.1.1 (R Foundation for Statistical Computing, Vienna, Austria).

### Data presentation in a synthetic format

The structure of the synthetic table (**Figure 2**) was co-created in collaboration between members of the Ethiopian and Ugandan team (FA, DF, DS), a senior author (ML), and two statisticians (LGC, IM), and sent for revision to the whole IMPULSE study group. Two researchers (LGC, DF) double checked the final structure against the structure of the PRISM Users’ Kit (Appendix S15 in the **Online Supplementary Document**). In the synthetic format (**Figure 2**), following the PRISM methodology [29], and in the absence of pre-defined composite EN-MINI indicators, indicators belonging to the same sub-domain were aggregated via averages to favour the inclusion of the highest amount of information, and provide a high-level overview of findings (Appendices S9–14 in the **Online Supplementary Document**).

## RESULTS

The sample included 51 sites (1 central MoH, 20 DHOs, and 30 facilities). Facilities represented different levels of care and both urban and rural settings (**Table 1**). Sample sizes varied by tool and site level, according to the EN-MINI protocol (Appendices S5 and S6 in the **Online Supplementary Document**).

**Table 1:** IMPULSE study sample characteristics (m = 51, 1 central MoH, 20 DHOs, 30 facilities) for Uganda, n (%)

Site type (n = 51)	
Central health data office – MoH	1 (1.9)
DHO	20 (39.2)
Third level of referral (national or regional) hospital	5 (9.8)
Second level of referral hospital	12 (23.5)
First level of referral health facility	13 (23.5)
**Region (n = 51)**	
Kampala City Administration	2 (3.9)
Karamoja	16 (31.4)
Lango	16 (31.4)
West Nile	17 (33.3)
**Setting – health facility only**	
Urban	13 (43.3)
Rural	17 (56.7)
**Managing authority – health facility only (n = 30)**	
Public	23 (76.7)
Private not-for-profit	7 (23.3)

DHO – district health office, IMPULSE – IMProving qUaLity and uSE of newborn indicators, MoH – Ministry of Health

### Availability of ENAP-recommended indicators in DHIS2

Assessment of ENAP-recommended indicators in DHIS2 revealed substantial gaps in indicator definition and standardisation. Of the 16 indicators assessed, fewer than half had a complete and exact definition encompassing all required numerator and denominator elements, with most gaps observed in numerator definitions (**Table 2**). These inconsistencies indicate that, even when indicators are reported, a lack of standardised definitions may limit comparability and interpretability across levels of the health system.

**Table 2:** IMPULSE study: availability of ENAP indicators in the electronic RHIS (DHIS2), Uganda

16 ENAP indicators	Type	Numerator	Denominator	Full indicator
Institutional maternal mortality ratio (per 100 000 deliveries)	Impact	No exact definition	≥1 definition exact	≥1 definition exact
Stillbirth rate in a health facility	Impact	≥1 definition exact	≥1 definition exact	≥1 definition exact
Pre-discharge neonatal mortality rate	Impact	≥1 definition exact	≥1 definition exact	≥1 definition exact
Low birth weight among livebirths (%)	Impact	≥1 definition exact	≥1 definition exact	≥1 definition exact
Preterm birth (facility based)	Impact	≥1 definition exact	≥1 definition exact	≥1 definition exact
Caesarean section rate	Outcome	≥1 definition exact	≥1 definition exact	≥1 definition exact
Postnatal care for women (facility-based)	Outcome	No exact definition	No exact definition	No exact definition
Postnatal care for newborns (facility-based)	Outcome	No exact definition	≥1 definition exact	No exact definition
Newborns breastfed within one hour of birth	Outcome	≥1 definition exact	≥1 definition exact	NA
Newborn resuscitation with bag and mask​	Outcome	≥1 definition exact	≥1 definition exact	NA
Premature (LBW) babies initiating KMC​	Outcome	≥1 definition exact	NA	NA
Newborns treated for neonatal sepsis/infection	Outcome	NA	≥1 definition exact	NA
Chlorhexidine cord cleansing	Outcome	NA	≥1 definition exact	NA
Antenatal corticosteroid use	Outcome	NA	No exact definition	NA
Newborns with documented birthweight	Outcome	≥1 definition exact	≥1 definition exact	NA
Uterotonic for prevention of post-partum haemorrhage	Outcome	No exact definition	≥1 definition exact	NA

DHIS2 – District Health Information System 2, ENAP – Every Newborn Action Plan, IMPULSE – IMProving qUaLity and uSE of newborn indicators, KMC – kangaroo mother care, LBW – low birth weight, NA – not available, RHIS – routine health information system

### RHIS performance across PRISM framework domains

The findings of the RHIS performance based on PRISM’s macro and sub-domains showed a mix of strengths and weaknesses, with a pattern for more weaknesses at facility level (**Figure 2**).

#### Outputs: data quality and information use

The domain of data quality showed good performance for the availability and completeness of the 10 core and available ENAP indicators at facility level, specifically for numerator data elements (range = 83–100%). Denominator data elements (total birth and live births) also showed high availability and completeness at the DHO level (93–94%). Conversely, availability and completeness of numerator data elements at DHO level were substantially lower (49–79%), except for early initiation of breastfeeding and uterotonics for prevention of postpartum haemorrhage (PPH), which ranged from 89% to 91%.

Despite good in availability and completeness, data accuracy was a major critical gap, with few sites having perfect accuracy (26–61%), and only 2/10 indicators (uterotonics to prevent PPH and live births) reaching satisfactory level when a 20% tolerance was applied (81% and 89%, respectively). Use of data use for decision-making was limited across system levels. At facility level, all assessed data use indicators fell below the 80% threshold, with use of data for performance review being the weakest area (21–69%). The DHOs demonstrated somewhat better performance (33–81%), but still showed important gaps; notable strengths at this level included dissemination of reports to administrators (95%) and production of reports or bulletins based on RHIS data (80%).

#### Input factors: organizational aspects

Key strengths and gaps related to influential factors with data quality and use were observed in organisational inputs (**Figure 2**). At central MoH level, almost all assessed elements related to governance and planning were present. Most DHOs reported adherence to quality improvement standards (92%), perceived strong organisational commitment to high-quality data (92%), and had staff available to compile and analyse data (100%). In contrast, several foundational resources necessary to guarantee a good data flow were frequently lacking.

Only a few of the sites possessed the minimum item bundle required for RHIS activities (22% of DHOs, 24% of facilities). Access to working internet connection was limited, particularly at the DHO level (60% of DHOs, 83% of facilities), and only half of DHOs reported having a staff capacity plan (55%). Another hindering factor to data quality assessed was the suboptimality of supervision practices, with limited availability of effective supportive supervision tools (68% of DHOs), irregular supervision schedules (only 64% of facilities reporting a supervision visit in the previous three months), and incomplete adherence to supervision quality criteria (79% of facilities meeting all five items).

Promotion of information culture from the organisation showed mixed performance specific to data quality and use. While overall promotion was satisfactory at the DHO level and slightly lower at the facility level, commitment to high-quality data was rated highly only at the DHO level (92%). In contrast, crucial data use related factor such as evidence-based decision-making was consistently the least promoted organisational practice, reported by 64% of DHO staff and 58% of facility staff.

#### Input factors: technical aspects

Technical capacity varied substantially by system level (**Figure 2**), suggesting how RHIS can impair staff capabilities in carrying out data quality and use tasks within specific settings. Data analysis and visualization skills were strong at DHO level with 85%-95% of staff capable of performing key tasks using the electronic RHIS (eRHIS). In contrast, facilities demonstrated weaker technical capacity, with fewer than 80% of staff able to perform most assessed tasks. Across both levels, the ability to calculate coverage indicators emerged as a common limitation, reported by 67% of DHO staff and only 47% of facility staff.

#### Input factors: behavioural aspects

Behavioural indicators also differed markedly by system level (**Figure 2**), while broadly following the trends described in the technical aspects. At the DHO level, most indicators showed high scores, with 87–97% of respondents reporting confidence and competence in performing RHIS-related tasks. An exception was staff motivation, with only 67% reporting feeling motivated to carry out RHIS duties. Behavioural performance at the facility level was lower and more heterogeneous, with indicator values ranging from 20% to 78%, suggesting a suboptimal level of staff preparedness in carrying out data quality and use of eRHIS-related tasks, in line with the previously discussed weaknesses. There was also a substantial gap between self-reported confidence and objectively assessed competence observed at the facility level, with differences reaching up to 48 percentage points (Appendix S16 in the **Online Supplementary Document**), underlying the relevance of possible lack of training and the suboptimal promotion of information culture at facility level previously discussed.

#### Process factor: data management

Data visualisation practices were widespread, reported by 95% of DHOs and 93% of facilities. Data management aspects related to data quality and use showed some major weaknesses in two major sub-domains. Only half of facilities (50%) and 74% of DHOs reported adequate data analysis practices. Similarly, timely feedback mechanisms were inconsistent, with feedback reports sent within a three-month period by 70% of DHOs and only 54% of facilities. These aspects suggest a lack of a functioning data management process and quality control, possibly hindering data quality.

### End users’ perspective

**Figure 3:**
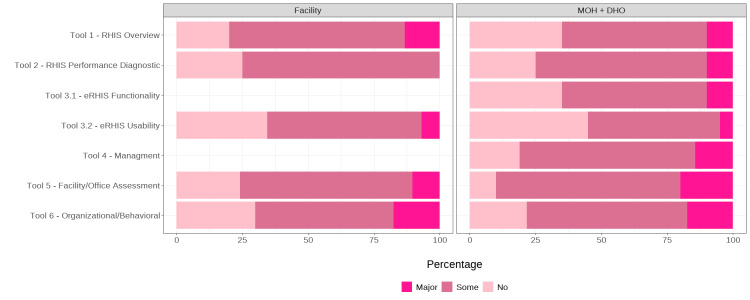
IMPULSE study: Ugandan end users’ perspectives on the need for improvement (350 respondents). DHO – district health office, eRHIS – electronic routine health information system, IMPULSE – IMProving qUaLity and uSE of newborn indicators, MoH – Ministry of Health, RHIS – routine health information system.

We collected 350 responses from end users: 144 from DHOs/MoH and 206 from facilities (Appendix S6 in the **Online Supplementary Document**). Overall, 74% of respondents reported a need for improvement, with no statistically significant differences between site levels (*P*-values >0.1). The highest demand for improvement among DHOs and MoH respondents was recorded for the domain of physical/human resources (80.9%) and behavioural/organisational aspects (90%) (**Figure 3**). Facility respondents reported more homogeneous needs across domains, with data collection processes and information system mapping and flow being the most frequently cited areas requiring improvement (80%).

## DISCUSSION

Here we assessed stillbirth and newborn data quality and use and underlying input and process factors within Uganda’s RHIS, and identified a consistent and policy-relevant pattern across system levels. A key finding is the marked divergence between high data availability and completeness, and persistently poor data accuracy, thus undermining data value and limiting data use for decision-making. Framed through the PRISM framework, this pattern shows that reporting compliance alone is insufficient, with technical, organizational, and behavioural factors shaping RHIS performance [19].

Similar discrepancies between reported data completeness and accuracy have been documented in Uganda and other LMICs [22,31–33]. While parallel reporting systems, inconsistent indicator definitions, and limited harmonisation of registers and DHIS2 outputs have been suggested as contributing factors [31–33], our findings point more strongly to systemic weaknesses in routine data verification and analytical capacity at the point of data generation. Moreover, the consistency of accuracy gaps across indicators and facility types suggests structural challenges, rather than isolated reporting errors.

Limited use of data for performance review and decision making emerged as a second major weakness, particularly at the facility level. Although DHOs demonstrated some strengths such as dissemination of reports and production of RHIS-based bulletins, routine use of newborn and stillbirth data to guide service improvement remained suboptimal. This finding aligns with previous evidence from Uganda and comparable contexts showing that access to RHIS data does not automatically translate into data-informed action [34]. Within the PRISM framework, this gap likely reflects constrained decision space at lower system levels, insufficient analytical skills, and weak accountability mechanisms linking data review to management action [34–36].

Our findings also highlight how organisational and resource-related factors further help explain the performance patterns. While governance and planning structures were strong at national and district levels, critical enabling resources such as minimum item bundles, reliable internet connectivity, and staff development plans were frequently lacking, particularly at the facility level. In addition, supervision practices were inconsistent in both frequency and quality. Similar resource and sustainability constraints have been documented elsewhere, especially in settings where RHIS strengthening relies heavily on external support [37]. These findings underscore the importance of embedding RHIS financing, capacity development, and supervision within routine district and national health system structures to ensure continuity and local ownership [38,39].

Behavioural and technical factors were more pronounced at facility level, where large gaps were observed between self-reported confidence and objectively assessed competence in RHIS tasks. This mismatch suggests that confidence alone is insufficient to ensure correct execution of more complex data tasks, such as calculating coverage indicators or interpreting trends. Comparable patterns have been reported in other studies examining RHIS performance and data use in sub-Saharan Africa [34,40]. These findings highlight the need for targeted, practice-oriented mentorship and supportive supervision, rather than reliance on one-off trainings or self-assessed competence [32,41,42].

End-user perspectives further highlighted the system-level constraints. Most respondents across all levels expressed a need for improvement, particularly in domains related to resources, data management, and organisational support. This is consistent with prior studies showing that improvements in data quality and use require coordinated action across the health system rather than isolated interventions [43,44].

From a policy and implementation perspective, our findings point to four priority areas for action. First, strengthening routine data verification and validation mechanisms particularly at facility level is essential to address persistent accuracy gaps. Second, building practical analytical capacity among frontline health workers, including skills for basic analysis, interpretation, and feedback, is critical to improving data use. Staff development plan and strategic resource allocation should be better utilised to bolster health workers capability in carrying out data related tasks. Third, institutionalising regular data review and performance dialogue within existing supervision and management structures may help bridge the gap between data availability and decision-making, in favour of a more broad and efficient data use. Evidence from DHIS2-focused initiatives suggests that such approaches can enhance both data quality and accountability when embedded in routine practice [45–47]. Lastly, physical resources required to maintain a functioning data flow such as internet connection and computers should be boosted, specifically at the facility level.

This study has several strengths, including the analysis of many indicators (>370) related to newborn and stillbirth data quality and use, the use of standardised EN-MINI Tools, reliance on direct observation, and application of a predefined analytical framework across multiple system levels. However, some limitations should be considered. The purposive selection of study sites limits national representativeness; nevertheless, the study approach is replicable and can be applied in other Ugandan settings. Furthermore, the over stratification of the results (*e.g.* facility type) could lead to loss of information. Future studies should focalise in setting specific results to better identify critical settings. Lastly, aggregation and averaging among indicators, as for the PRISM methodology may imply losing some differences, which we balanced by providing detailed results (**Online Supplementary Document**).

## CONCLUSIONS

The IMPULSE phase 1 findings from Uganda demonstrate that improving newborn and stillbirth data quality requires moving beyond reporting compliance to address the underlying determinants of accuracy and use. Therefore, by distinguishing between data availability, accuracy, and use, and by linking these outcomes to technical, organisational, and behavioural factors, this study provides actionable evidence to guide RHIS strengthening efforts. Future phases of IMPULSE and related implementation research should focus on testing targeted interventions addressing these priority gaps and assessing their sustainability over time.

## Additional material


Online Supplementary Document
